# Advancing Practices to Increase Access to Diabetes Self-Management Education and Support Through State Health Departments

**DOI:** 10.5888/pcd21.240255

**Published:** 2024-11-27

**Authors:** Melissa Bing, Robert Montierth, Bernadine Cabansay, Pollyanna Leung, Leslie Harrison

**Affiliations:** 1 Division of Diabetes Translation, National Center for Chronic Disease Prevention and Health Promotion, Centers for Disease Control and Prevention, Atlanta, Georgia; 2 Oak Ridge Institute For Science and Education, Oak Ridge, Tennessee; 3 School of Pharmacy, University of California San Francisco

Currently, 38 million Americans have diabetes, a complex and chronic condition that is the leading cause of adult kidney failure, adult blindness, and lower-limb amputations (1). Learning how to manage this condition is a crucial skill for people with diabetes (PWD). The cornerstone of diabetes management is diabetes self-management education and support, or DSMES, which aims to provide PWD with the “knowledge, skills, and confidence” needed for good self-care (2). The benefits of DSMES are vast and include clinical outcomes such as improved hemoglobin A1c levels and behavioral outcomes including enhanced self-efficacy and problem-solving skills to manage diabetes. Despite these advantages, DSMES remains widely underused (2).

In 2018, the Centers for Disease Control and Prevention (CDC)’s Division of Diabetes Translation and the Division for Heart Disease and Stroke Prevention launched DP18–1815 (1815), *Improving the Health of Americans Through Prevention and Management of Diabetes and Heart Disease and Stroke.* This 5-year cooperative agreement focused on diabetes management and type 2 diabetes prevention, as well as heart disease and stroke prevention, and was awarded to the state health departments (SHDs) of each of the 50 states and the District of Columbia, to run from June 29, 2018, to June 30, 2023 (3).

One of the initiatives encouraged in 1815 was to improve access to and participation in American Diabetes Association (ADA)–recognized or Association of Diabetes Care and Education Specialists (ADCES)–accredited DSMES services in underserved areas. All 51 recipients worked on this initiative, and in this essay we reflect on several activities related to engaging the pharmacy sector, establishing umbrella organizations, and engaging in continuous quality improvement (Table). These activities were identified through 1815 deliverables, such as recipient progress reports. Detailed descriptions of work related to DSMES were extracted from the deliverables and coded for analysis.

**Table:** Examples of Activities Implemented by State Health Departments to Increase Access to Diabetes Self-Management Education and Support, 2018–2023

State health department	Example activities
**Texas**	The Texas Department of State Health Services worked with the University of Texas College of Pharmacy to develop a “Pharmacy-Led DSMES Guide” to aid in the delivery and sustainability of DSMES in pharmacy settings.
**Oklahoma**	The Oklahoma State Department of Health supported amendments to include pharmacists as reimbursable providers of DSMES by Oklahoma State Medicaid.
**North Carolina**	The North Carolina Department of Health and Human Services became a DSMES umbrella sponsor before 1815. Under 1815, they scaled up the number of subsites and provided technical assistance on an array of topics.
**Maryland**	The Maryland Department of Health partnered with the School of Pharmacy at Notre Dame of Maryland University to establish DSMES umbrella organizations to increase the region’s access to DSMES services.
**Michigan**	The Michigan Department of Health and Human Services collaborated with a large health care system in metro Detroit to pilot a social needs screener for persons with diabetes referred to DSMES services.
**Colorado**	The Colorado Department of Public Health and Environment worked with health systems to identify people with diabetes, restructuring electronic health record systems and workflows for efficient scheduling, screening, and documentation, with referrals primarily between clinics and DSMES providers.

Abbreviation: DSMES, diabetes self-management education and support.

## Engaging the Pharmacy Sector

During 1815, SHDs played a significant role in supporting pharmacies in advancing DSMES offerings nationwide. Approximately 73% of SHDs engaged with community- and ambulatory care–based pharmacists in their respective jurisdictions to provide DSMES start-up information, training, and ongoing technical assistance to establish pharmacy-based DSMES services. Given the treatment complexities related to successfully managing diabetes, pharmacists were recognized as valuable partners of the health care delivery team based on their medication expertise, ease of access when questions from PWD arise, and presence in nearly every US community (4). The ADA endorses pharmacists as integral providers of DSMES services, leading many SHDs to pursue strategic partnerships with the pharmacy sector (2).

As the partnerships progressed, barriers to successfully sustaining DSMES in pharmacy practice locations became evident. A lack of experience with DSMES billing in pharmacies and low participation rates due to the nontraditional DSMES setting posed challenges to a sustainable pharmacy-based DSMES model (5). Recipients responded as follows to address barriers and improve sustainability: 1) developed promotional materials to increase awareness of DSMES among PWD and health care providers (74%); 2) strengthened referral pathways for health care providers to refer PWD to pharmacy-based DSMES programs (16%); 3) provided tailored technical assistance for pharmacists at DSMES sites (16%); and 4) aided in the establishment of a DSMES billing infrastructure, such as implementing pharmacy billing support mechanisms (10%). Additional support from SHDs included integrating community health workers to promote pharmacy-based DSMES programs (10%) and aiding in exploring and developing a multi-site network of pharmacy-based DSMES programs (2%).

Support from SHDs provided pharmacists with the necessary tools to lead innovations and lay the groundwork for potential improvements in delivering DSMES. Innovative opportunities that use the pharmacy infrastructure in a turn-key manner to efficiently scale DSMES services within pharmacy networks and chains would be of great value. SHDs have a vested interest in increasing the capacity of the pharmacy sector to succeed by not only increasing the availability of DSMES within communities but also by helping to strengthen and sustain quality DSMES services.

## Umbrella Organizations

Recipients of 1815 regularly cited administrative challenges as a key barrier to increasing DSMES access. The umbrella organization approach is a successful method for alleviating several of these challenges and establishing sustainable reimbursement. It allows a sponsor organization to become ADA-recognized or ADCES-accredited and then help subsites become certified under their umbrella certification. The umbrella sponsor can support the subsites with administrative components such as completing the accreditation or recognition process, reporting requirements for the certifying body, and helping with billing and reimbursement.

Throughout the 1815 funding period, 25% of recipients pursued a DSMES umbrella organization approach. Some recipients worked on a model where the SHD served as the umbrella sponsor to establish a centralized system that allowed them to provide support to DSMES partners (22%). Support included submitting applications and data for certification and providing technical assistance to DSMES services on an array of services ranging from billing to curriculum usage. States that applied this approach were able to establish subsites at locations ranging from local health departments to health care practices to community pharmacies.

Two states pursued the umbrella organization approach by establishing sponsor organizations at universities or regional health care institutions. This format enabled the sponsors to offer many of the same benefits as an SHD-based sponsor but within a smaller jurisdiction.

## Continuous Quality Improvement

Continuous quality improvement (CQI) efforts in health care may be critical to advancing and adapting in an ever-changing environment. Defined as a “progressive incremental improvement of processes, safety, and patient care,” CQI is generally considered a successful tool in improving health care (6). Under the 1815 cooperative agreement, 55% of recipients leveraged CQI approaches to advance DSMES efforts. Common CQI efforts involved increasing awareness and referrals to DSMES services by health care providers (60%) as well as advancing patient awareness of DSMES and improving enrollment (66%). CQI efforts were implemented in various settings, including health care systems, pharmacies, and local health departments, using common methods such as “plan–do–study–act,” process maps, workflow analyses, and decision trees. The most successful CQI efforts among recipients spanned multiple years, allowing for multiple CQI cycles. They targeted specific processes, including diabetes diagnosis, referral form completion, follow-up notifications, laboratory referral workflow, and educational opportunities.

States’ CQI activities identified several key findings, including:

1. PWD referred to DSMES services regularly cited transportation as the most significant barrier to DSMES attendance, prompting 17% of DSMES providers to explore telehealth platforms;
2. PWD needed to be contacted up to 4 times via multiple modes of communication (eg, text, phone calls, electronic health record [EHR] platforms) to begin the DSMES intake process, resulting in 33% of health care systems reformatting their referral process and incorporating multiple outreach efforts; and
3. There was a lack of familiarity of both DSMES and how to make referrals, prompting SHDs to provide ongoing training for health care providers on the benefits of DSMES and successfully navigating the referral system using existing EHR systems (31%).

## Future Implications

SHDs can be essential in coordinating chronic disease prevention and management efforts at the population level. This is evident in CDC’s continued investment in a new cooperative agreement, DP23–0020 (2320), *A Strategic Approach to Advancing Health Equity for Priority Populations with or at Risk for Diabetes.* This cooperative agreement will extend from June 30, 2023, to June 29, 2028, and funds the 50 SHDs, the District of Columbia, and 26 local and national organizations to implement diabetes management and type 2 diabetes prevention strategies with a focus on priority populations (7). Under 2320, more than 84% of recipients (n = 65) have selected to work on a strategy to improve access, appropriateness, and feasibility of DSMES services through a health equity lens.

Engaging the pharmacy sector has been a key approach for 2320 recipients to advance DSMES access and participation. As highly accessible and trusted health care professionals, pharmacists can play a pivotal role in ensuring that diabetes programming, including DSMES, is both accessible and appropriate for priority populations. SHDs can further support the pharmacy sector in strengthening screening processes and community referrals for identified social determinants of health (8).

Recipients of 2320 are also working to establish and sustain new DSMES umbrella organizations in regions lacking DSMES. By strategically locating newly established sponsors or subsites in areas accessible to priority populations, such as within a network or pharmacy chain, these umbrella organizations can help bridge gaps and improve participation in DSMES among communities that need it most. Additionally, umbrella organizations strengthen the individual DSMES site by enhancing service offerings, sharing resources, and supporting long-term sustainability.

Those interested in scaling these approaches can apply them within many geographic and health care settings. Umbrella organizations can also exist in nontraditional health care settings, such as health departments. Throughout 1815, the effectiveness of these approaches was gauged by the number of DSMES sites within a state and the number of PWD that attended DSMES. A more robust evaluation should be tailored to the approach and incorporate additional data points, such as rates of DSMES referrals and enrollment and successful billing encounters.

Although the approaches in this essay are described in relation to diabetes management, they may also be applicable to other chronic disease programs. For example, the umbrella approach can be seen in the emergence of community care hubs, which support and provide a range of evidence-based chronic disease management and prevention programs hosted by community-based organizations and that feature a centralized hub for joint administrative and operational functions (9). The unique structure of the umbrella and community care hub model allows staff and organizations to have specialized roles. For example, staff within the sponsor entity may serve as billing specialists, increasing the likelihood of reimbursement, while staff at the subsites are able to allocate more effort to program referrals and delivery. Recipients of 2320 are also exploring this tailored version of the umbrella model.

Ultimately, applying the 3 approaches described in this essay may help ensure PWD have equitable access to DSMES and an opportunity for improved diabetes-related outcomes.
